# MicroRNA-509-3p inhibits cellular migration, invasion, and proliferation, and sensitizes osteosarcoma to cisplatin

**DOI:** 10.1038/s41598-019-55170-2

**Published:** 2019-12-13

**Authors:** Sagar L. Patil, Asha Palat, Yinghong Pan, Kimal Rajapakshe, Rachna Mirchandani, Maria Bondesson, Jason T. Yustein, Cristian Coarfa, Preethi H. Gunaratne

**Affiliations:** 1 Department of Pediatrics, Columbia University, New York, NY USA; 2 Department of Biology and Biochemistry, University of Houston, Houston, TX USA; 3 Department of Molecular and Cellular Biology, Baylor College of Medicine, Houston, TX USA; 4 Department of Intelligent Systems Engineering, Indiana University, Bloomington, IN USA; 5 Texas Children’s Cancer and Hematology Centers, Department of Pediatrics, Baylor College of Medicine, Houston, TX USA

**Keywords:** Bone cancer, Tumour heterogeneity, miRNAs

## Abstract

Osteosarcoma (OS) is the most common primary pediatric malignancy of the bone having poor prognosis and long-term survival rates of less than 30% in patients with metastasis. MicroRNA-509 was reported to be downregulated in OS. We and others previously published that miR-509-3p can strongly attenuate cellular migration/invasion and sensitize ovarian cancer to cisplatin. Here, we show that overexpression of miR-509-3p inhibited migration of primary OS cell lines U2OS, HOS, and SaOS2 as well as metastatic derivatives 143B and LM7. miR-509-3p overexpression also inhibited proliferation and invasion of HOS and 143B cells and sensitized cells to cisplatin. Luciferase reporter assays using 3′-UTRs of predicted miR-509-3p targets associated with metastatic phenotypes revealed ARHGAP1 could be one of the downstream effectors of miR-509-3p in HOS. To find the global impact of miR-509-3p overexpression and cisplatin treatment we performed Reverse Phase Protein Analysis (RPPA). AXL, which has been reported to play a critical role in cisplatin resistance and confirmed as direct target of miR-509-3p was downregulated upon miR-509-3p treatment and further down-regulated upon miR-509-3p + cisplatin treatment. We propose that the miR-509-3p/AXL and miR-509-3p/ARHGAP1 axes have the potential to uncover new druggable targets for the treatment of drug resistant metastatic osteosarcoma.

## Introduction

Osteosarcoma (OS), which arises primarily in children and adolescents, is the most common primary malignancy of the bone and the most frequent cause of cancer-related death in children^1,2^. Currently, combination chemotherapy consisting of doxorubicin, cisplatin and methotrexate, along with surgery is used to treat OS^1^. The 5-year survival is nearly 75% for patients with no evidence of metastatic disease at diagnosis in contrast to 30% for patients who present with gross evidence of metastatic lesions on radiographic imaging^3^. Approximately 20% of the OS patients present with metastases and ~50% of patients who experience OS recurrence develop metastasis within 18 months after chemotherapy^3^. Pulmonary metastasis is the most common site and most common cause of death of OS patients^3,4^. Systemic chemotherapy has increased long-term survival rates up to nearly 75% in patients with localized OS, but has had a minimal impact on overall survival of patients with metastatic disease^5,6^. New approaches targeting metastasis-specific cellular pathways therefore have been a major focus^7^. However, unlike cancers with reciprocal chromosomal translocations, OS has complex karyotypes with multiple genetic alterations^8^. Whole-genome sequencing of tumors from 32 OS patients showed that cancer-specific TP53 rearrangements were found in more than 50% of patients^9^.

MicroRNAs are small ~22 nucleotide long endogenous non-coding RNAs that have important regulatory roles in post-transcriptional gene silencing of hundreds of targets within and between diverse cellular pathways^10^. microRNA-509 (miR-509-5p), was reported to be downregulated, in OS tissues and cell lines^11^. This observation also suggests that miR-509-3p, which is transcribed along with miR-509-5p, is downregulated in OS tissues. Previously, we reported that miR-509-3p is a strong tumor suppressor that attenuates migration and disrupts multi-cellular spheroids in multiple ovarian cancer (OVCA) cell lines^12^. In the p53-mutant ovarian cancer line OVCAR8, we established YAP1 to be a direct downstream target and a critical effector of miR-509-3p-mediated tumor suppression^12^. YAP1 is highly expressed in both human and mouse OS and knockdown of YAP1 was found to reduce OS tumor progression in mouse models^13^. We therefore, hypothesized that the miR-509-3p/YAP1 axis can be used to identify and develop new druggable targets for metastatic OS.

In this study, we investigated the role of miR-509-3p in migration and invasion of primary and metastatic OS cell lines. Cisplatin is one of the most commonly used drugs for the treatment of OS^14,15^. Cisplatin has been reported to inhibit migration and invasion of ovarian cancer^16^. We examined the role of miR-509-3p in migration and invasion of OS cells in the presence or absence of the chemotherapeutic drug cisplatin and through microRNA target predictions and Reverse Phase Protein Array (RPPA) we identified downstream effectors of miR-509-3p.

Our study found that miR-509-3p overexpression inhibited OS migration and invasion and sensitized OS to cisplatin. Downregulation of direct targets ARHGAP1 and AXL in miR-509-3p treated OS cell lines and further downregulation of AXL upon miR-509-3p + cisplatin treatment suggests that the miR-509-3p/AXL axis could reveal genes and pathways that drive cisplatin resistance in OS.

## Results

### miR-509-3p inhibits OS migration

**Figure 1:**
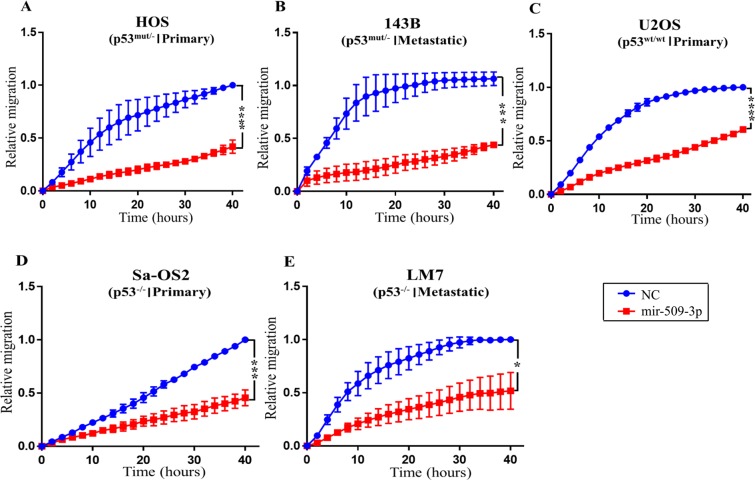
miR-509-3p inhibits OS migration. *In vitro* 96 well plate scratch/wound healing assay after transiently overexpressing miR-509-3p mimic and scrambled negative control RNA (NC) in (**A**) HOS (p53^mut/−^, primary) and its metastatic derivative (**B**) 143B (p53^mut/−^); (**C**) U2OS (p53^wt/wt^, primary); (**D**) Sa-OS2 (p53^−/−^, primary) and it’s metastatic derivative (**E**) LM7 (p53^−/−^) cell lines. (N ≥ 3). Migration at all time points was normalized to migration at the final time point (40 hours) of cells treated with NC to obtain relative migration. Error bars represent standard errors of mean (SEM) between biological replicates. Time course starts ~72 h post transfection. P values were calculated at 24 h intervals between NC and miR509-3p treated cells (*p ≤ 0.05, **p ≤ 0.01), (***p ≤ 0.001, ****p ≤ 0.0001).

To assess ability of miR-509-3p to inhibit OS migration we performed 96 well plate scratch/wound healing assays on five OS cell lines including primary HOS (p53^mut/−^) and its metastatic derivative 143B (p53^mut/−^); primary SaOS2 (p53^−/−^) and it metastatic derivative LM7 (p53^−/−^); and primary U2OS (p53^wt/wt^) cell line (Fig. 1). Transient overexpression of miR-509-3p mimc strongly inhibited migration in all five cell lines as compared to a negative control scrambled RNA (NC) transfected cells. At the 24 h time point, the relative migration of HOS transfected with miR-509-3p mimc was 0.23 units (arbitrary units relative to NC at the final time point) compared to 0.77 units in NC (P = 0.004) (Fig. 1A). The relative migration of 143B (p53^mut/−^) cells transfected with miR-509-3p mimc was 0.28 units compared to 1.00 units in NC treatment (P = 0.004) (Fig. 1B). The relative migration of U2OS (p53^wt/wt^) cells transfected with miR-509-3p mimc was 0.35 units as compared to 0.91 units in NC (P = 9.39e^−005^) (Fig. 1C). Primary SaOS2 (P53^−/−^) cells with null p53 (slow growing and less metastatic), migrated slower than the other four cell lines, and miR-509-3p overexpression further inhibited its migration. The relative migration of SaOS2 cells transfected with miR-509-3p mimc was 0.27 units compared to 0.58 units in NC (P = 0.004) (Fig. 1D). The relative migration of LM7 (P53^−/−^) cells transfected with miR-509-3p mimc was 0.38 units compared to 0.89 units in NC (P = 0.011) (Fig. 1E). Collectively these results demonstrate that miR-509-3p inhibits OS cell migration irrespective of p53 status or metastatic capability of OS.

### ARHGAP1 is a direct downstream target of miR-509-3p and a critical effector of miR-509-3p-mediated migration inhibition

**Figure 2:**
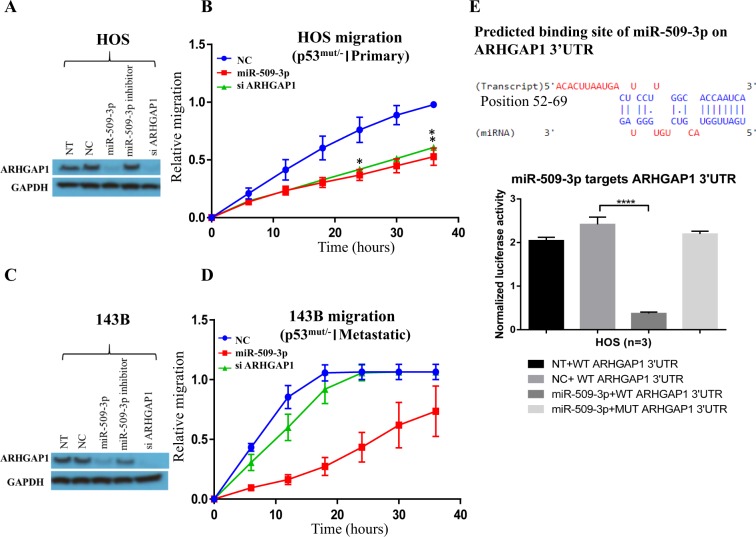
ARHGAP1 is a critical effector of miR-509-3p-mediated migration inhibition. (**A)** Western blot analysis of ARHGAP1 protein level in HOS cells, 72 h post transfection of miR-509-3p mimic and NC. (**B)** *In vitro* 96 well plate scratch plate/wound healing assay in HOS cells. (**C)** Western blot analysis of ARHGAP1 protein level in 143B cells, 72 h post transfection of miR-509-3p mimic and NC. Blots presented here are cropped from same blot and full-length blots are presented in Supplementary Fig. 3. (**D)** *In vitro* 96 well plate scratch plate/wound healing assay in 143B cells and (**E**) TargetScan prediction of miR-509-3p binding site on 3′UTR of ARHGAP1 and ARHGAP1 luciferase reporter activity for the predicted binding site, 48 h post transfection of miR-509-3p mimic and NC into HOS cells.

To elucidate downstream effectors of miR-509-3p-driven attenuation of migration in OS cells, we used siRNAs against genes that were predicted targets of miR-509-3p, and were associated with ECM and cell adhesion (Supplementary Fig. 1A). In our previous work, we established that YAP1 is a direct downstream target and a critical effector of miR-509-3p in the p53-mutated ovarian cancer cell line OVCAR8^12^. In contrast to OVCAR8 cells, we found that siRNA to YAP1 had little effect on HOS and 143B cell migration (Supplementary Fig. 1A). By sharp contrast, knockdown of another predicted target gene, ARHGAP1, by siRNA inhibited HOS cell migration to the same extent as miR-509-3p mimic (Fig. 2B and supplementary Fig. 1A). The relative migration of HOS cells transfected with siARHGAP1 was 0.41 units (P = 0.034) and the relative migration of HOS cells overexpressing miR-509-3p was 0.37 units (P = 0.030) compared to 0.76 units in NC at 24 h (Fig. 2B).

Overexpression of miR-509-3p in OS cell lines resulted in downregulation of mRNA and protein levels of ARHGAP1 (Fig. 2A and Supplementary Fig. 1C,D). To confirm actual binding of miR-509-3p to its predicted binding site on 3′-UTR of ARHGAP1 gene (Fig. 2E), we performed a luciferase reporter assay in HOS cells. Luciferase signal was significantly repressed and this repression was lost in HOS cells carrying a luciferase reporter with mutations in the 3′-UTR of ARHGAP1 (Fig. 2E). This confirms that ARHGAP1 is a direct target of miR-509-3p.

Interestingly, siARHGAP1 had no impact on migration of the metastatic 143B cell line even though Western blots confirmed that overexpression of miR-509-3p led to down-regulation of ARHGAP1 protein in both HOS and 143B cells, which had almost equal amounts of ARHGAP1 (Fig. 2A–D). Relative migration of 143B cells treated with siARHGAP1 was 1.00 units and relative migration of cells treated with miR-509-3p was 0.43 units compared to 1.00 units in NC at 24 h (Fig. 2D).

### miR-509-3p inhibits invasion and proliferation of HOS and 143B cell lines and sensitizes cells to cisplatin

**Figure 3:**
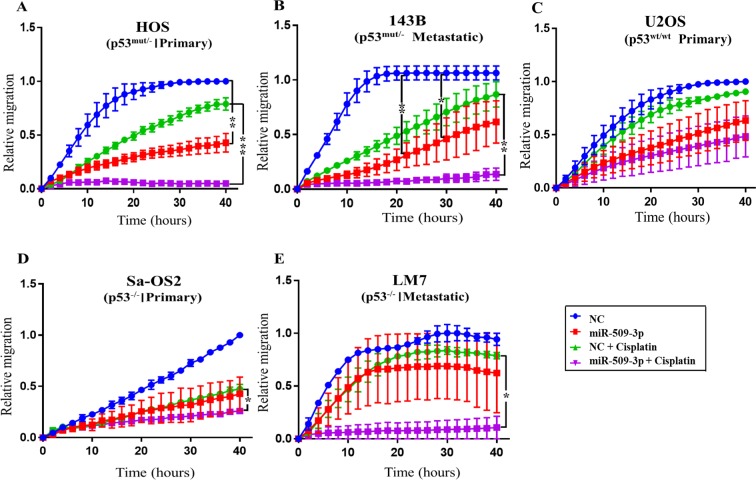
Cellular migration assays performed on 5 OS cell lines treated with miR-509-3p mimic + cisplatin. *In vitro* 96 well plate scratch plate/wound healing assay were performed after transiently overexpressing miR-509-3p mimic and NC with and with and without cisplatin (2.5 ug/ml). The cell lines tested include (**A)** HOS (p53^mut/−^, Primary) and its metastatic derivative (**B)** 143B (p53^mut/−^); (**C)** U2OS (p53^wt/wt^, Primary); (**D)** Sa-OS2 (p53^−/−^, Primary) and its metastatic derivative (**E**) LM7 (p53^−/−^) cell line. (N ≥ 3 except LM7 N = 2). Migration at all time points was normalized to migration at final time point (40 h) of cells treated with NC to get relative migration. Error bars represent SEM between biological replicates. Time course starts ~72 h post transfection. P values are calculated at 24 hour between NC and miR-509-3p and between NC + cisplatin and miR-509-3p + cisplatin treated cells (*p ≤ 0.05, **p ≤ 0.01), (***p ≤ 0.001, ****p ≤ 0.0001).

Cisplatin is one of the most commonly used drugs for the treatment of OS^14,15^. Cisplatin has been reported to inhibit migration and invasion of ovarian cancer^16^. To test the impact of the combination of miR-509-3p and cisplatin, we performed 96-well plate scratch/wound healing assay with all five OS cell lines transfected with miR-509-3p in combination with cisplatin treatment (2.5 µg/ml) (Fig. 3). Combination of miR-509-3p and cisplatin treatment (miR-509-3p + cisplatin) completely inhibited migration of the primary cell line HOS (p53^mut/−^) and its metastatic derivative 143B (p53^mut/−^), and LM7 (p53^−/−^), a metastatic derivative of SaOS2 compared NC, miR-509-3p alone or NC with cisplatin (NC + cisplatin) (Fig. 3A,B,E). In HOS cells relative migration was 0.04 units in miR-509-3p + cisplatin treatment, 0.33 units in miR-509-3p treatment and 0.56 units in NC + cisplatin treatment compared to 0.94 units in NC at 24 h. In the 143B cell line, relative migration was 0.08 units in miR-509-3p + cisplatin treatments, 0.34 units in miR-509-3p treatment and 0.57 units in NC + cisplatin treatments compared to 1.06 units in NC at 24 h. In the LM7 cell line, relative migration was 0.08 units in miR-509-3p + cisplatin treatments, 0.68 units in miR-509-3p treatments and 0.82 units in NC + cisplatin treatments compared to 0.92 units in NC at 24 h. Combination of miR-509-3p and cisplatin treatment had little effect on cell migration of primary cell line U2OS (p53^wt/wt^) and Sa-OS2 (p53^−/−^) compared to cells treated with miR-509-3p alone (Fig. 3C,D). In U2OS cell line, relative migration was 0.34 units in miR-509-3p + cisplatin treatments, 0.42 units in miR-509-3p treatment and 0.76 units is in NC + cisplatin treatments compared to 0.90 units in NC at 24 h. In the Sa-OS2 cell line, relative migration was 0.18 units in miR-509-3p + cisplatin treatments, 0.29 units in miR-509-3p treatment and 0.29 units in NC + cisplatin treatments compared to 0.56 units in NC at 24 h. We conclude that a combination of miR-509-3p transfection and cisplatin treatment reduced cell migration more efficiently than either compound alone in OS cell lines, although to a varying degree in the different cell lines.

**Figure 4:**
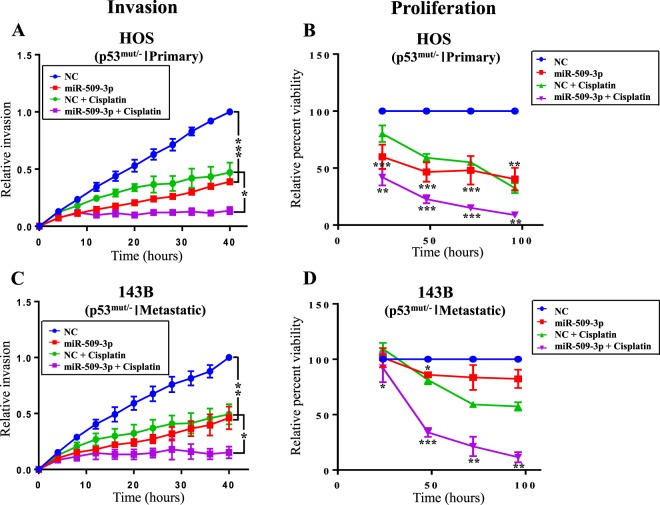
miR-509-3p inhibits OS invasion and proliferation, and sensitizes OS to cisplatin. *In vitro* 96 well plate Matrigel invasion assay after transiently overexpressing miR-509-3p mimic and NC with and with and without cisplatin (2.5 ug/ml) in (**A**) HOS and (**C**) 143B cells. (N = 3). Invasion at all time points was normalized to invasion at final time point (40 h) of cells treated with NC to get relative invasion. Error bars represent SEM between biological replicates. Time course starts ~72 h post transfection. Cells proliferation assay (MTS assay) after transiently overexpressing miR-509-3p mimic and NC with and without cisplatin (2.5 ug/ml) in **B**) HOS and **D**) 143B cells. (N = 3). P values are calculated at 24, 48, 72 and 96 h between NC and miR-509-3p and between NC + cisplatin and miR-509-3p + cisplatin treated cells (*p ≤ 0.05, **p ≤ 0.01), (***p ≤ 0.001, ****p ≤ 0.0001).

In order to study the effect of miR-509-3p on other cancer related phenotypes in OS, we focused on the primary HOS cell line and its metastatic derivative 143B. To test the impact of miR-509-3p on invasion and proliferation of HOS and 143B cells, we performed Matrigel invasion and proliferation assays (Fig. 4). miR-509-3p strongly inhibited invasion and proliferation of HOS cells compared to NC (Fig. 4A,B). The combination of miR-509-3p and cisplatin (2.5 µg/ml) had an even greater impact on inhibiting invasion and proliferation of HOS cells (Fig. 4A,B). In HOS cells the relative invasion was 0.12 units in miR-509-3p + cisplatin treatments, 0.23 units in miR-509-3p treatment and 0.36 units in NC + cisplatin treatments compared to 0.62 units in NC at 24 h (Fig. 4A). In HOS cells relative percent viability was 8.75% in miR-509-3p + cisplatin treatments, 40.32% in miR-509-3p treatment and 32.43% in NC + cisplatin treatments compared to 100% in NC treated cells at 96 h (Fig. 4B).

miR-509-3p significantly inhibited invasion in 143B cells, but had only a moderate effect on proliferation compared to NC (Fig. 4C,D). The combination of miR-509-3p + cisplatin (2.5 µg/ml) had the largest impact on inhibiting invasion and proliferation of 143B cells (Fig. 4C,D). In 143B cells, the relative invasion was 0.14 units in miR-509-3p + cisplatin treatments, 0.27 units in miR-509-3p treatment and 0.37 units in NC + cisplatin treatments compared to 0.67 units in NC at 24 h. In 143B cells, relative percent viability was 11.50% in miR-509-3p + cisplatin treatments, 57.45% in miR-509-3p treatment and 82.32% in NC + cisplatin treatments compared to 100% in NC at 96 h.

### RPPA revealed AXL down-regulation as one of the modes of miR-509-3p mediated cisplatin sensitivity

**Figure 5:**
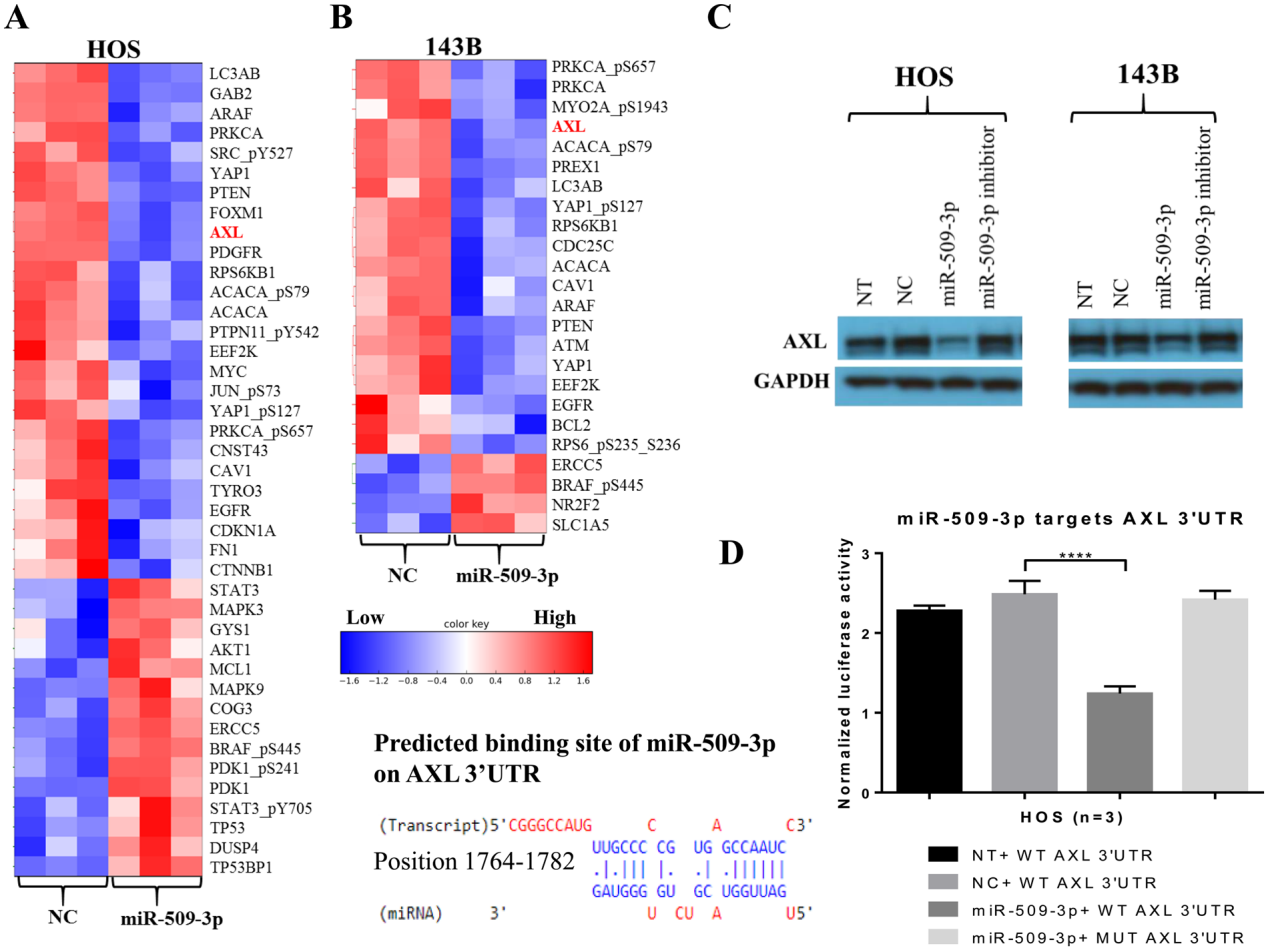
miR-509-3p downregulates AXL expression. Heat map depiction of proteins differentially expressed between NC and miR-509-3p overexpression in (**A**) HOS and (**B**) 143B cells. Protein expression was assessed by reverse phase protein array (RPPA). (**C**) Western blot analysis of AXL protein level, 72 h post transfection in HOS and 143B cells. Blots presented here are cropped from same blot and full-length blots are presented in Supplementary Fig. 3. (**D**) TargetScan prediction of miR-509-3p binding site on 3′UTR of AXL and AXL luciferase reporter activity for the predicted binding site, 48 h post transfection of miR-509-3p mimic and NC into HOS cells.

**Figure 6:**
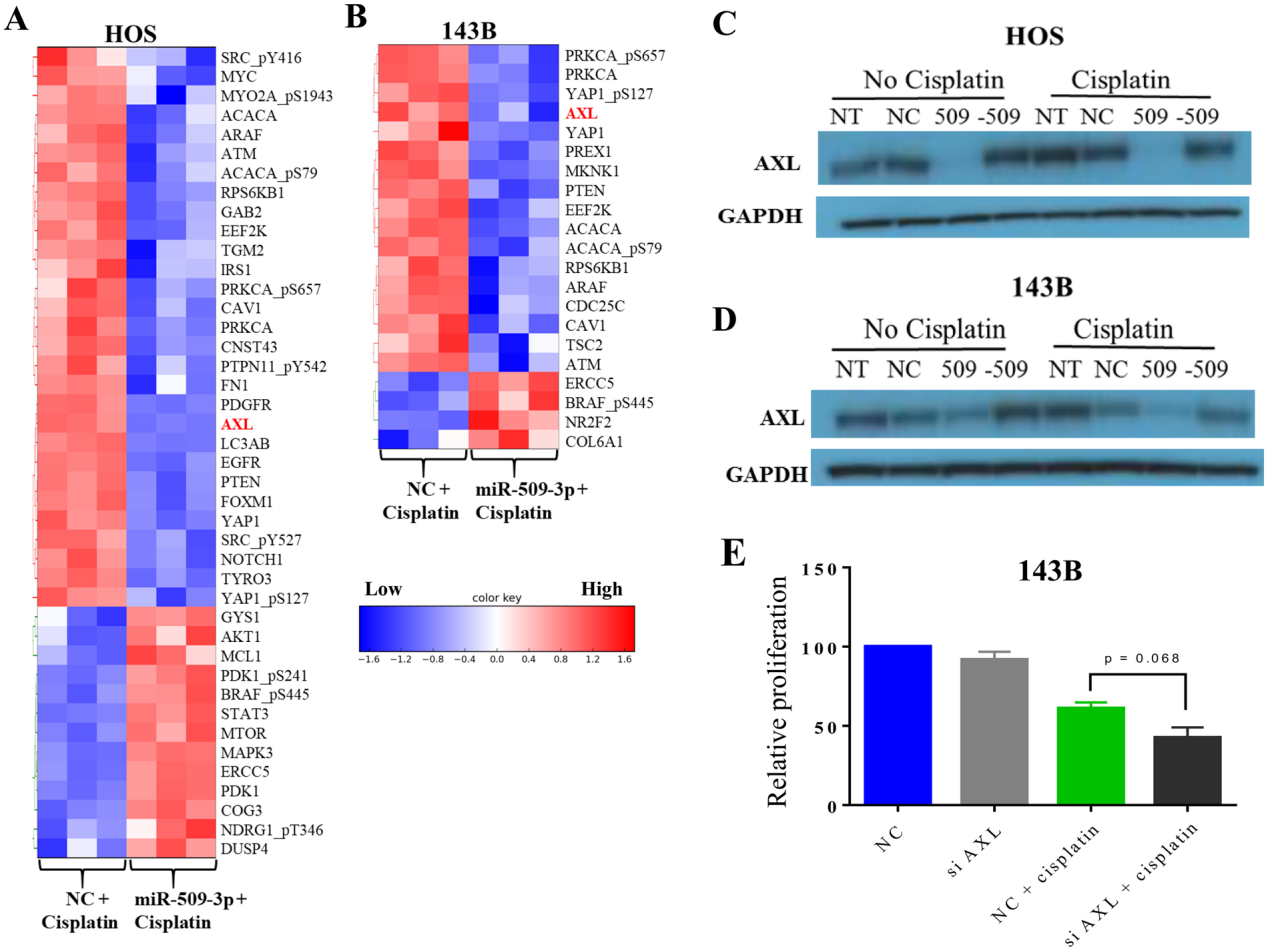
RPPA analysis on HOS and 143B treated with miR-509-3p+ cisplatin, and effect of siAXL + cisplatin on 143B. Heat map depiction of proteins differentially expressed between NC and miR-509-3p overexpression in (**A)** HOS and (**B)** 143B cells with and without cisplatin treatment. Western blot analysis of AXL protein level, 72 h post transfection of NT, NC, miR-509-3p (509), and miR-509-3p inhibitor (−509) in (**C)** HOS and (**D**) 143B cells with and without cisplatin treatment. Blots presented here are cropped from same blot and full-length blots are presented in Supplementary Fig. 4. (**E)** Proliferation assay (MTS assay) in 143B cells transfected with NC and siAXL with and without cisplatin at 72 h post transfection. (N = 3, except E, N = 2). Error bars represent SEM between biological replicates. P values calculated between NC + cisplatin and siAXL + cisplatin treated cells (*p ≤ 0.05, **p ≤ 0.01), (***p ≤ 0.001, ****p ≤ 0.0001).

To uncover downstream effectors of miR-509-3p action in HOS and 143B cells, we performed reverse phase protein array, in which the expression levels of 296 different proteins involved in cancer development and progression were measured after miR-509-3p mimic or control transfections. We observed that in HOS and 143B cells, overexpression of miR-509-3p mimic resulted in a reduced AXL protein expression compared to NC (Fig. 5A,B). Earlier reports have shown that downregulation of AXL inhibits 143B migration and invasion^8,17^. Western blot analysis showed that the AXL protein was downregulated in miR-509-3p treated 143B cells (Fig. 5C). We confirmed AXL to be a direct target of miR-509-3p by luciferase reporter assay (Fig. 5D). To uncover critical downstream effectors of miR-509-3p + cisplatin combination, we compared protein expression changes between miR-509-3p + cisplatin and NC + cisplatin treated cells. We observed that AXL, which is known to drive acquired resistance to cisplatin, were downregulated in both HOS and 143B cell lines treated with miR-509-3p + cisplatin compared to NC + cisplatin treatments (Fig. 6A–D)^18–21^. The combination of siAXL and cisplatin decreased proliferation to 42.6% (Fig. 6E). In addition to AXL, other genes such as ATM involved in cisplatin resistance and which are not direct target of miR-509-3p were also downregulated in miR-509-3p + cisplatin treatment. This suggests that miR-509-3p sensitizes OS cells to cisplatin partly by directly downregulating AXL and partly by indirectly downregulating other genes involved in cisplatin resistance.

## Discussion

miR-509-3p, expressed from a ~100 kb genomic cluster of miRNAs on Xq27.3, is emerging as a strong tumor suppressor in relation to multiple cancer types. Zhang *et al*. 2017, reported that miR-509-5p is downregulated in OS which suggests that miR-509-3p, which is expressed on the same hairpin as miR-509-5p is also downregulated in OS. miR-509-3p has been established to inhibit proliferation and increase sensitivity to cisplatin in chemo-resistant ovarian cancer cells and influence the cell cycle, colony formation and migration of human epithelial lung and cervical cancer cell line^12,22,23^. Here we report that miR-509-3p is also a strong tumor suppressor of OS, in which it inhibits cellular migration, invasion and proliferation and also sensitizes OS cells to cisplatin. Although previous work from our laboratory, established YAP1 to be a direct downstream target and a critical effector of miR-509-3p-driven inhibition of migration in OVCAR8^12^, we found that this is not the case for OS cells. siRNA to YAP1 had no effect on HOS and 143B cell migration (Supplementary Fig. 1A,B). This is likely because microRNAs act through different genetic networks in cells of different genetic backgrounds. Instead, we found that ARHGAP1 (CDC42 GTPase-Activating Protein1) which is also associated with ECM and cell adhesion to be a direct downstream target and critical effector of miR-509-3p-mediated inhibition of migration in HOS cells. ARHGAP1 is also a direct target of miR-34a and downregulation of ARHGAP1 alone was sufficient to inhibit human lung adenocarcinoma invasion^24^. Interestingly, we found that although miR-509-3p was able to downregulate ARHGAP1 expression in both HOS and 143B cells the ability of siARHGAP1 to inhibit migration and invasion was limited to HOS cells. HOS and 143B are derived from the same patient however 143B has Ras stable expression. We propose that this and other mutations that may have accumulated during metastatic progression of HOS may have allowed 143B cells to circumvent the dependence on ARHGAP1 and utilize alternate pathways for driving cellular migration. This observation adds to the reports from us and other groups that, microRNAs can have different phenotypic impacts in different cell types even within same tumor type.

In order to uncover downstream effectors of miR-509-3p in the metastatic derivative 143B cell line, we performed RPPA and observed that AXL was among the genes that were significantly downregulated following miR-509-3p treatment of both HOS and 143B cells. Knockdown of AXL in 143B cells was previously reported by others to inhibit colony formation, migration, and invasion^8^. Another microRNA, miR-199a-3p, was reported to directly target AXL to inhibit 143B cell migration and invasion^17^. AXL has been linked with chemoresistance in a number of different cancer types^18–20,25^. In addition to AXL other proteins such as ATM which are also implicated in chemo-resistance^21,26^ were also found to be downregulated in cells treated with miR-509-3p and cisplatin which suggest that AXL downregulation could be one of the many events that lead to chemo-sensitivity in OS cells. AXL is a receptor tyrosine kinase that plays an important role in the progression of cancer by enhancing proliferation and migration, and inhibiting apoptosis and therapeutic resistance^25^.

AXL receptor signaling suppresses p53 in melanoma through stabilization of the MDMX–MDM2 complex and AXL inhibition is shown to increase the expression of p53 target genes and sensitize cells to cisplatin^27^. We found that miR-509-3p overexpression caused downregulation of AXL and upregulation of p53 (Supplementary Fig. 2). This suggests that AXL-induced modulation of the MDMX–MDM2 hetero-complex stability can suppress p53 activity, which in turn desensitizes cells to cisplatin in melanoma^27^. Most importantly, AXL is frequently phosphorylated (P-AXL) in OS patient samples^8^. Activated AXL (P-AXL) is highly expressed in OS cells, and this expression significantly correlates with the recurrence and lung metastasis of OS patients^28^. Furthermore, high expression of P-AXL is predictor for worse prognosis in patients with OS^28^.

As per our knowledge, this is the first report of confirming ARHGAP1 and AXL as a true direct target of miR-509-3p. We have successfully demonstrated that both of these miR-509-3p targets were independently identified and validated in our osteosarcoma models and have significant biological ramifications. Further mechanisms downstream of ARHGAP1 and AXL can be explored in future which can give better understanding of role of these genes in OS metastasis and chemo-resistance. Based on GeneCards (https://www.genecards.org/), AXL and ARHGAP1 together are associated with the Actin nucleation (important for membrane trafficking, leading edge protrusion during cell migration, cell division etc.) and GPCR signaling (involved in a wide variety of physiological processes, including being involved in growth and metastasis of some types of tumors.) pathways. Based on the network from GeneMania (https://genemania.org/), AXL and ARHGAP1 both interact with PIK3 isoforms – PIK3R1 and PIK3R2 indicating a connection to the PI3K/Akt pathway. In future regulatory relations/functions between AXL and ARHGAP1 can be studied to gain more insight into complex molecular pathways regulated by microRNAs.

**Figure 7:**
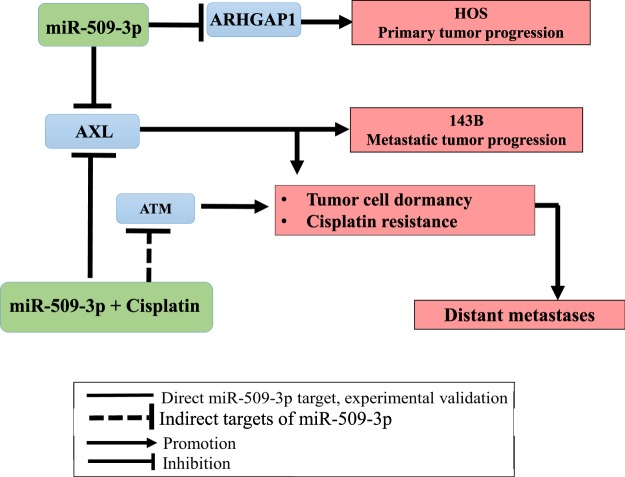
Model for miR-509-3p-mediated inhibition of migration/invasion and sensitization to cisplatin in OS. miR-509-3p directly inhibits ARHGAP1 in primary OS cell line HOS and AXL in metastatic OS cell line 143B to inhibit migration and invasion. miR-509-3p elicits sensitivity to cisplatin partially by directly inhibiting AXL and partially by indirectly inhibiting ATM.

Our working model for how miR-509-3p inhibits OS migration, invasion and proliferation by targeting the ARHGAP1 and sensitizes OS cells to cisplatin in part by downregulating AXL is outlined in Fig. 7. Systemic chemotherapy, which, increases long-term survival rates up to 75% in patients with localized OS has had only a minimal impact on the overall survival of patients with metastatic disease^5,6^. We propose that the miR-509-3p/AXL and miR-509-3p/ARHGAP1 axes could be used to discover new druggable targets to significantly reverse chemo-resistance and inhibit cellular migration/invasion of cell from the primary tumor, thus leading to improved clinical outcomes for patients presenting with metastatic OS.

## Methods

### Cell lines and growth conditions

OS cell lines HOS, 143B, U2OS, SaOS2, and LM7 were provided by Dr. Jason T. Yustein, Department of Pediatrics, Baylor College of Medicine, Houston, Texas with an MTA in place. All OS cell lines were cultured in DMEM medium (Thermo Fisher Scientific, Waltham, MA, USA) and supplemented with 10% heat-inactivated fetal bovine serum (FBS) (Atlanta Biologicals, Flowery Branch, GA, USA). All the cultures were maintained at 37 °C in a humidified incubator with 5% CO_2_.

### Transient transfection/overexpression of microRNA and siRNA

Cells were seeded at 100,000 cells per well in a 6-well culture plate, or 4,000 cells per well in a 96-well culture plate (for proliferation assay), or 580,000 cells per 100 mm culture dish (for protein extraction). After 24 h, cells were transfected with microRNA or siRNA by using Lipofectamine 2000 or 3000 reagent (Thermo Fisher Scientific, Waltham, MA, USA), following manufacturer’s recommended protocol. Details of micorRNA and siRNA as follows; NC (cat # 4464073); miR-509-3p (cat # 4464067), miR-509-3p inhibitor (cat # 4464084), siARHGAP1 (cat # 4392420), siYAP1 (cat # 4392420), siRAC1 (cat # 4390824), siEGFR (cat # AM16708) (all from Thermo Fisher Scientific, Waltham, MA, USA) and custom made siAXL (5′ AGAUUUGGAGAACACACUGA 3′) (Dharmacon, Lafayette, CO, USA). Three to four hours post transfection, Opti-MEM with 10% FBS was added to each well at a volume equal to the transfection reaction volume. Twenty four hours post transfection, the transfection reaction was removed and fresh DMEM with 10% FBS was added.

### Total RNA isolation

Total RNA was isolated at indicated time points using miRNA Easy Mini Kit (Qiagen, Venlo, Netherlands). Cells were washed twice with PBS, and harvested by centrifugation. Cell pellets were lysed using Qiazol lysis buffer (Qiagen, Venlo, Netherlands). Total RNA including the miRNA fraction was isolated following the manufacturer’s instructions. The purity and concentration of the extracted RNA was determined using an ND-1000 Nanodrop spectrophotometer (Thermo Fisher Scientific, Waltham, MA, USA)

### Reverse transcription and quantitative Real Time PCR (qRT-PCR/qPCR) for mRNAs

One microgram of total RNA from each sample was reverse transcribed using the high-capacity cDNA reverse transcription kit (Applied Biosystems, Foster City, CA, USA) according to the manufacturer’s instructions. Reverse transcription was carried out with the Veriti Thermal Cycler system (Applied Biosystems, Foster City, CA, USA). All qPCR experiments were performed using Power SYBR Green Master Mix (Applied Biosystems, Foster City, CA, USA) on StepOnePlus Real-Time PCR System (Applied Biosystems, Foster City, CA, USA) and analyzed manually using the ΔΔCt method. 18 S or GAPDH were used as endogenous controls for all targets. All qPCR reactions were performed in three technical and at least three biological replicates.

### Reverse transcription and quantitative Real Time PCR (qRT-PCR/qPCR) for microRNAs

Ten nanograms of total RNA was reverse transcribed in 15 µl final reaction volume using the respective stem-loop RT primers with the TaqMan microRNA reverse transcription kit (Applied Biosystems, Foster City, CA, USA) according to the manufacturer’s instructions. All qPCR experiments were performed using a Taqman Universal qPCR Master Mix without AmpErase UNG (Applied Biosystems, Foster City, CA, USA) on StepOnePlus™ Real-Time PCR System (Applied Biosystems, Foster City, CA, USA) and analyzed manually using the ΔΔCt method. RNU48 was used as an endogenous control. All RT-PCR reactions were performed in three technical and at least four biological replicates.

### Scratch plate/wound healing assay

Scratch plate/wound healing assays were performed using the IncuCyte ZOOM Live-Cell Analysis System (Essen BioScience, Ann Arbor, MI, USA). Sixty hours post transfection, cells were collected from six well plates and seeded in 96-well ImageLock microplates (Essen BioScience, Ann Arbor, MI, USA) at a cell density of 35,000 cells per well. Cisplatin was added (2.5 µg/ml) at the time of seeding in appropriate wells. Twelve hours after seeding (72 h after transfection), artificial scratch wounds were made using the WoundMaker™ (Essen BioScience, Ann Arbor, MI, USA). Each well was washed twice with PBS and then overlaid with 200 μl of DMEM medium supplemented with 10% FBS. Images at different time points were acquired using the IncuCyte-ZOOM™ live cell imaging system. Cell migration was calculated with IncuCyte’s automated image analysis algorithm using the Relative Wound Density (RWD) metric. A single experiment included 3 technical replicates and RWD at each time point was normalized to RWD at the final time point (40 h) of scrambled negative RNA control (NC) to obtain relative migration. Average relative migration between independent experiments was plotted as a function of time. At least three biological replicates were used to plot relative migration. Student t-test was used for calculating statistical significance. Error bars represent standard error of mean (SEM) between biological replicates.

### Luciferase reporter assay

To generate the plasmid with the wild-type target binding sequence, a region of about 500 bps, containing the predicted microRNA binding site was PCR amplified from human genomic DNA and cloned into the NotI restriction site of a psiCHECK-2 vector (Promega, Madison, WI, USA) using the Gibson Assembly Cloning Kit (New England Biolabs, Ipswich, MA, USA). For ARHGAP1, it was a 492 bp long DNA fragment (−1438 to −1930 nt) with the predicted miR-509-3p target site located at −1898 to −1905 bp in the ARHGAP1 3′-UTR. For AXL, a 501 bp long DNA fragment (−3585 to −4085 nt) with the predicted miR-509-3p target site located at −4034 to −4040 bp in the AXL 3′-UTR was utilized. Mutant clones were generated by mutating the miR-509-3p seed interaction site in the reporter plasmid construct, using the Q5 Site-Directed Mutagenesis kit (New England Biolabs, Ipswich, MA, USA). For ARHGAP1-mut (ACCAATCA- > ACtAAgCA) and for AXL-mut (GCCAATC- > GCgAtTC). The clones were verified by Sanger sequencing. HOS cell line was plated at 10,000 cells/well in a 96-well plate. One hundred nanograms of the wild-type reporter plasmid was transfected alone (NT), or co-transfected with 20 nM of either a scrambled negative RNA control (NC) or a miR-509-3p mimic. To confirm the reduction in luciferase was due to direct binding of the microRNA to the 3′UTR, a fourth experimental condition was included where cells were transfected with one hundred nanograms of the mutant plasmid and 20 nM of the miR-509-3p mimic. Transfections were performed using Lipofectamine 2000 (Life Technologies, Carlsbad, CA, USA) following the manufacturer’s recommended protocol. Luciferase assays were performed at 48 h post transfection using a Dual-Luciferase Reporter Assay System (Promega, Madison, WI, USA). Renilla luminescence signals were normalized by the firefly luciferase signals. Three biological replicates were performed for each assay.

### Cell proliferation/viability assay

Cell proliferation/cell viability was assessed by MTS assay. In a 96 well plate, 4000 cells per well were seeded in DMEM media with 10% FBS. After 24 h, cells were transfected with miR-509-3p mimic, NC, and appropriate siRNAs. At the indicated time points (24 h, 48 h, 72 h and 96 h), media from the 96-well plate was removed and 100 µl pre-warmed DMEM media with 10% FBS was added. After 15-20 mins, 20 μl of CellTiter 96® AQueous One Solution Cell Proliferation Assay (MTS) (Promega, Madison, WI, USA) was added to each well and the plate was incubated in the dark at 37 °C for 1 h as recommended by the manufacturer. Absorbance was recorded at 490 nm using SpectramaxM5 plate reader (Molecular Devices, San Jose, CA, USA). Absorbance was normalized to absorbance from scrambled RNA control (SC/NC) transfected wells. Percent viability was calculated by the following formula (Absorbance of treatment well/absorbance of NC well) X 100. Average of biological replicates was plotted with SEM. Student’s t test was performed to calculate statistical significance.

### Matrigel invasion assay

Cell invasion assays were performed using Matrigel-coated 96-well microplates. Plates were coated overnight with 50 µl of 0.1 mg/ml Matrigel matrix (BD Biosciences, San Jose, CA, USA), before seeding 35,000 cells per well in 96 well plate cell culture plate. Scratch/wounds were generated as described for the scratch-wound migration assay. After making scratch and washing wells, 50 µl of Matrigel matrix, diluted to 1 mg/ml in cell culture medium, and was added to each well. Microplates were incubated at 37 °C to set the Matrigel and were overlaid with 200 μl of culture medium 24 h later. Images at different time points were acquired using the IncuCyte-ZOOM™ live cell imaging system. Density was plotted as a function of time for three independent experiments, with error bars showing SEM between biological experiments. Student’s t-test was used for calculating statistical significance.

### Reverse phase protein array

In a six-well cell culture plate, 100,000 cell per well were seeded 24 h before transfection. Cells were treated with various treatments as described. Cells were harvested at 72 h after transfection washed twice with PBS and lysed using RPPA lysis buffer as recommended by MD Anderson Cancer Center Core Facility, Houston, TX. Cellular protein concentration was determined by Bradford assay. Protein concentration was adjusted to 1.5 µg/ml. The cell lysate was mixed with 4X SDS Sample Buffer with freshly added β-mercaptoethanol (β-Me) as recommended by MD Anderson Cancer Center Core Facility, Houston, TX. Samples were boiled for 5 min, and stored in −80 °C until sample submission. RPPA with the 295 standard antibody list used by MD Anderson Cancer Center Core Facility was performed at MD Anderson Cancer Center Core Facility, Houston, TX.

### Western blots

Cells were treated as described for a period of 72 h and were collected and pelleted. The cell pellets were washed with PBS twice. The supernatant was discarded and the cell pellets were stored at −80 °C until protein extraction. Protein extraction was performed using RIPA buffer (Sigma-Aldrich, St. Louis, MO, USA) with ProBlock^TM^ protease inhibitor cocktail (GoldBio, St Louis, MO, USA) and Simple Stop phosphatase inhibitor cocktail (GoldBio, St Louis, MO, USA). Fifty micrograms of protein per well were size separated on NuPAGE 4-12% Bis-Tris gels using Bolt^TM^ MOPS SDS buffer (Invitrogen, Carlsbad, CA, USA). The proteins were transferred to PVDF membranes (Invitrogen, Carlsbad, CA, USA) and blocked for an hour at room temperature with a 5% solution of non-fat dry milk dissolved in TBST (50 mM Tris-HCl, 150 mM NaCl, 0.1% Tween 20, pH 7.5). Primary antibodies for ARHGAP1 (Abcam, Cambridge, MA, USA, cat # ab154338), AXL (Cell Signaling Technology, Danvers, MA, USA, cat # 8661) and GAPDH (Cell Signaling Technology, Danvers, MA, USA, cat # 5174) were added at a concentration of 1:2000 in 5% bovine serum albumin (Sigma-Aldrich, St. Louis, MO, USA, cat # A9647) and incubated overnight at 4 °C. Secondary HRP-linked anti-rabbit antibodies (Cell Signaling Technology, cat # 7074) were added at a concentration of 1:20,000 in 5% dry milk in TBST and incubated for an hour at room temperature. Proteins were finally visualized using SuperSignal^TM^ West Femto Maximum Sensitivity Substrate (Thermo Fisher Scientific, Waltham, MA, USA, cat # 34095).

## Supplementary information

Supplementary information

## Data Availability

All data produced during the current study are included in this article and its supplementary files.
