# Supplementary material for: MicroRNA-509-3p inhibits cellular migration, invasion, and proliferation, and sensitizes osteosarcoma to cisplatin
Source: Sci Rep. 2019 Dec 13;9:19089. doi: 10.1038/s41598-019-55170-2 (PMC6911094; doi:10.1038/s41598-019-55170-2)
Supplement: Supplementary file 1 — Supplementary information [file 41598_2019_55170_MOESM1_ESM.pdf]

MicroRNA-509-3p inhibits cellular migration, invasion, and proliferation, and sensitizes osteosarcoma to cisplatin.

Sagar L. Patil[1], Asha Palat[1], Yinghong Pan[1], Kimal Rajapakshe[4], Rachna Mirchandani[1], Maria Bondesson[2], Jason T. Yustein[3], Cristian Coarfa[4], and Preethi H. Gunaratne[1]*.

Supplementary figure 1

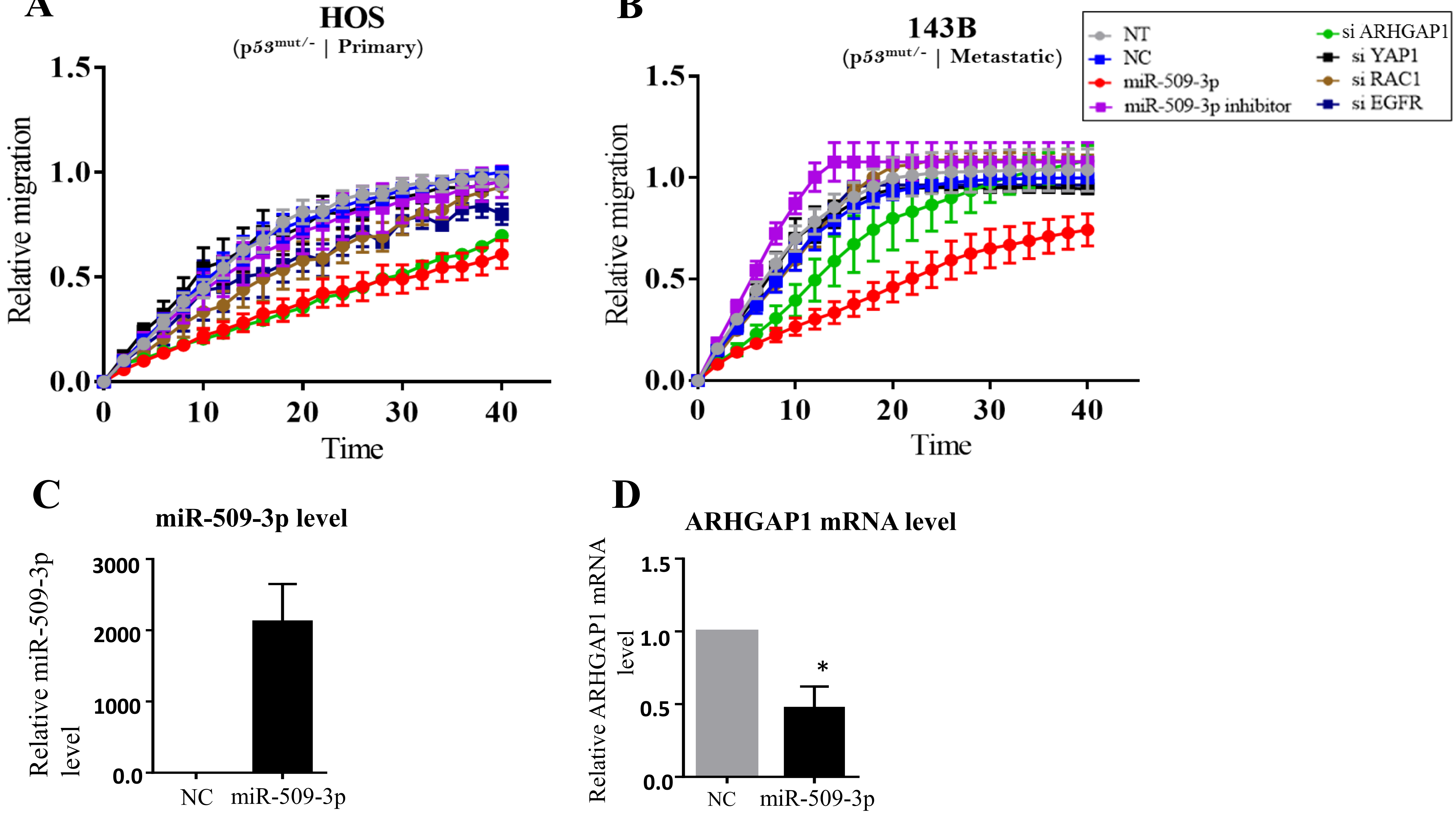

Supplementary figure 1. Cellular migration assays performed on OS cell lines treated with siRNAs targeting 4 potential downstream targets. *In vitro* 96 well plate scratch plate/wound healing assays were performed after transiently overexpressing miR-509-3p mimic, scrambled negative control RNA (NC), siYAP1, siRAC1, siARHGAP1, and siEGFR. The cell lines tested included A) HOS ($p53^{mut/-}$, Primary) and its metastatic derivative B) 143B ($p53^{mut/-}$). Time course starts ~72 h post transfection. **C)** Relative miR-509-3p and **D)** ARHGAP1 transcript level at 72 h post transfection of miR-509-3p mimic and NC in HOS cells.

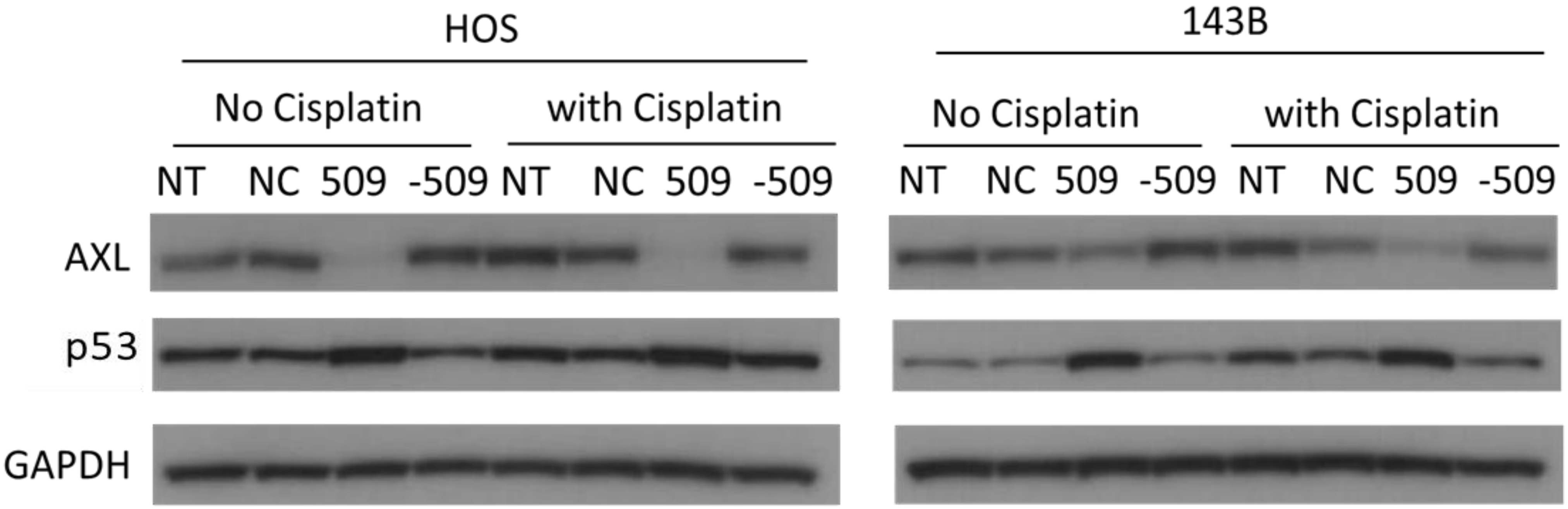

Supplementary figure 2. Western blot analysis of AXL and p53 72 h post miR-509-3p transfection in HOS and 143B cells. Blots presented here are cropped from same blot and full-length blots are presented in Supplementary figure 4.

Supplementary figure 3

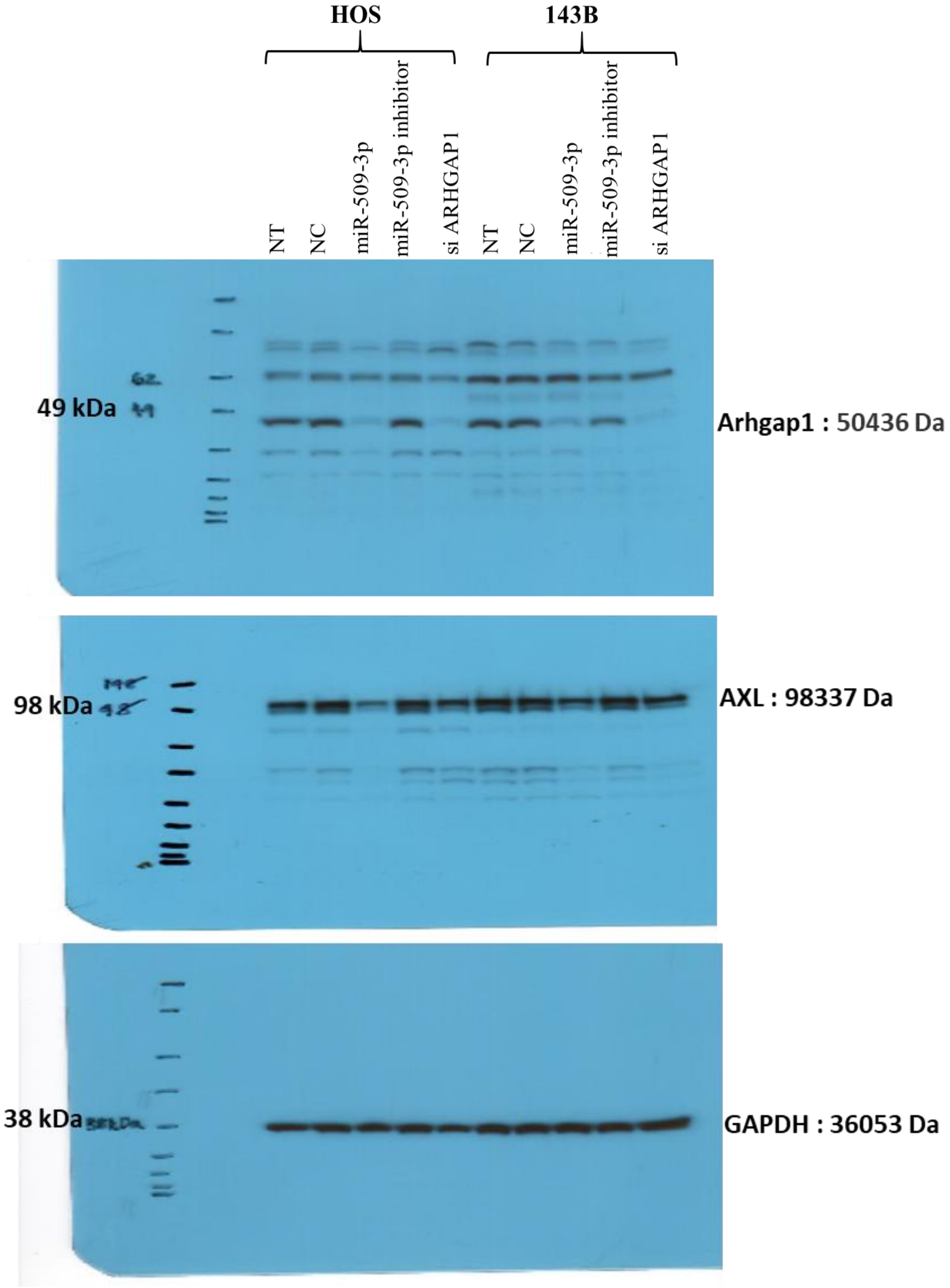

Supplementary figure 3: Original unaltered full-length western blots used in fig. 2A, 2C and fig. 5C.

Supplementary figure 4

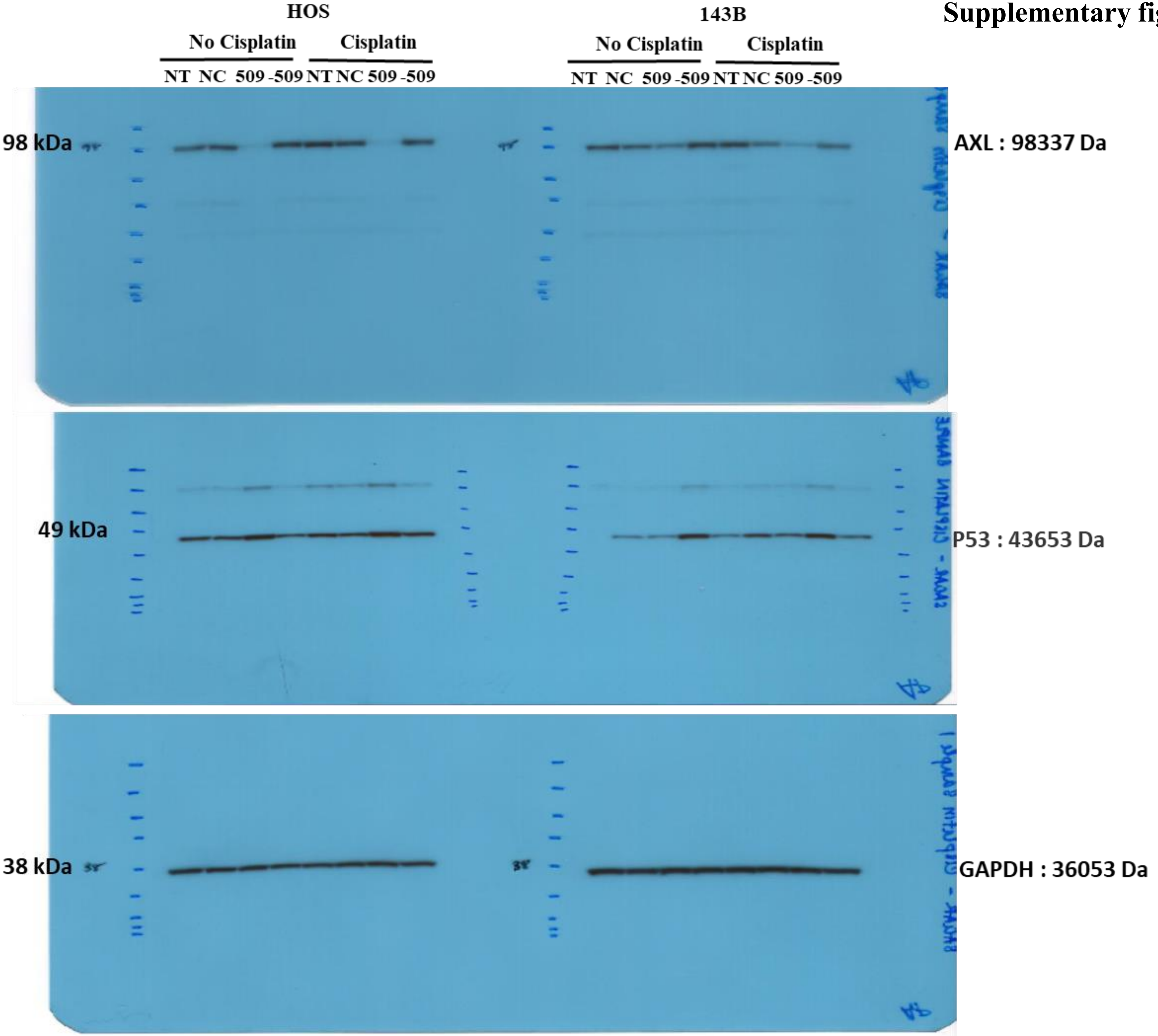

Supplementary figure 4: Original unaltered full-length western blots used in fig. 6C, 6D and supplementary fig 2.
